# Revisiting Mechanism of NaOH Dechlorination Treatments for Bronze Conservation in Quantitative Study

**DOI:** 10.3390/ma17246126

**Published:** 2024-12-14

**Authors:** Xin Yang, Wei Wu, Kunlong Chen

**Affiliations:** 1 Institute for Culture Heritage and History of Science and Technology, University of Science and Technology Beijing, Beijing 100083, China; d202410776@xs.ustb.edu.cn; 2 Key Laboratory of Archaeomaterials and Conservation, Ministry of Education, Beijing 100083, China; 3 Shanghai Key Laboratory of Material Protection and Advanced Material in Electric Power, Shanghai University of Electric Power, Shanghai 200090, China

**Keywords:** quantitative study, simulated sample, NaOH dechlorination treatments, dechlorination endpoint

## Abstract

Dechlorination is a crucial strategy for archeological bronze stabilization to resist corrosion induced by cuprous chloride (CuCl). Conventional samples, either archeological or simulated ones, have deficiencies in revealing dechlorination mechanisms for their complex rust layers and difficulties in quantifying chlorine content. In this work, samples with fixed chlorine amounts were prepared by compressing method to solve overcomplicated and unquantifiable problems. Then, patina profiles and desalinization solutions were analyzed to revisit the dechlorination mechanism across varying solution concentrations and current densities after dechlorination treatments. Results indicate that the sodium hydroxide (NaOH) desalinization method is achieved by converting CuCl to trihydroxychloride (Cu_2_(OH)_3_Cl). However, this transformation leads to an expansion of the CuCl layer, nearly doubling the CuCl layer thickness at the current density of 25 μA/cm^2^. Dechlorination solution measurements provide information on quantifying chlorine removal and dechlorination progress. Theoretically, the endpoint (c_0_) for the NaOH dechlorination method is supposed to be a chloride ion concentration of 358.2 ppm. As the NaOH solution concentrations vary from 10^−6^ to 10^−2^, CuCl dechlorination progress (E_t=24h_) calculations are at about 3% to 6% at 24 h. Applying the current significantly improves the effectiveness of dechlorination at 2.5 μA/cm^2^. However, the chloride ion concentration in the solution starts to decrease after reaching a current density of 12.5 μA/cm^2^, even dropping to 12.07 ppm at 25 μA/cm^2^. According to a theoretical analysis, chlorine evolution during electrolytic processes would be responsible for this phenomenon.

## 1. Introduction

Cuprous chloride (CuCl, nantokite) is the principal agent of bronze disease. Once excavated, ancient bronze faces threats from unstable CuCl hydrolysis and oxidation reactions [1,2,3]. The corrosion product mainly involves cuprous oxide (Cu_2_O, cuprite) and copper trihydroxychloride (Cu_2_(OH)_3_Cl, atacamite, clinoatacamite, paratacamite, and botallackite) [4]. Hydrogen and chloride ions are released into patina and attack the well-conserved bronze matrix, in which CuCl and soluble chlorides accumulated in the rust layer threaten the stability of bronze [1,2,5,6]. As an effective conservation strategy, dechlorination removes chlorides from objects and further hinders secondary corrosion [7]. However, the limited availability of artifact samples and challenges in quantifying of accelerated-corrosion samples have resulted in inadequate research on quantifying dechlorination effectiveness and revealing mechanisms. Therefore, understanding these mechanisms in a quantitative way is essential for establishing a robust theoretical and practical foundation for the protection of archeological bronze.

Alkaline desalination methods are well-established and widely used techniques. In the early 20th century, Rathgen [8] already introduced this approach in conservation. By the 1970s, research conducted by North [9,10] established a solid foundation for further studies, investigating how factors such as solution concentration, pH, time, and temperature impact on the desalination of iron artifacts. During this period, some research suggested utilizing other alkaline solutions, such as LiOH and Na_2_CO_3_·NaHCO_3_, for desalination [11,12]. Sodium sesquicarbonate at 5% (*w*/*v*) in water slowly removes copper from the sound metallic regions of an object as well as from the corrosion crust [13,14]. In recent decades, studies focused on refining alkaline desalination technical parameters to improve efficiency and protective effects [15,16]. For example, electrochemical dechlorination methods based on alkaline soaking were commonly employed in practice because desalination efficiency was taken into consideration during operation [17]. This approach typically takes alkaline as a base electrolyte, applying an external electric field to enhance efficiency. After electrochemical treatment for chloride removal, further corrosion was inhibited by this process [18]. Although the number of articles on alkaline dechlorination increased, there is still a lack of progress in understanding its mechanism. As Bryce [19] and Rees-Jones [20] pointed out, cultural relics may exhibit a relatively fragile state or crack after desalination treatment; yet, no reasonable explanation or relevant evidence has been provided.

Desalination studies relied on archeological or corrosion simulated samples [13,16,18,21,22,23,24,25,26]. However, archeological samples are challenging for comparative studies on properties before and after treatments due to their complex and non-uniform rust layers. Accelerated-corrosion simulated samples help mitigate this problem to some extent. For example, Kim [16] dipped copper and bronze plates in 0.1 M HCl for 26 h to form CuCl, rusted with RH 75% for the formation of corrosion products. This method provides better consistency compared to archeological ones because similar corrosion environments prompt much more uniform corrosion product composition. However, this kind of chlorine-introduced method would lead to variable amounts and complex chemical states. Thus, in evaluating desalination effectiveness, it has to be measured by periodically examining the chlorine ion concentration in the solution [13,17,23,26]. Zhu [23] prepared samples by soaking them in a 3.5% NaCl solution for a week. During dechlorination treatment, a quantitative study was realized by measuring chlorine ion concentration from the desalting solution changed daily. However, the total amount of chloride removed and dechlorination progress were unable to be quantified accurately. Some researchers undertook some strategies to quantify the total chloride amount, but still faced some problems. Ouyang [17] calculated total chlorine removal but was unable to identify its source, from soluble chlorate or CuCl, because an extra source of chloride was introduced into patina of accelerated-corrosion samples. As a result, these methods struggled to provide accurate estimates of chlorine ion removal from the patina layer and establish a reasonable dechlorination endpoint.

Conventional dechlorination studies have difficulties in revealing corresponding mechanisms. Some tried to investigate in a quantitative way but found it still difficult to distinguish the source of chloride ions, whether from CuCl or other substances. If the removal of chloride ions from CuCl remains at a lower level, objects still face secondary corrosion risks. Therefore, it is essential to develop a more quantitative method to systematically investigate the transformation of corrosion products, dechlorination effectiveness, and influencing factors. This study, based on the NaOH desalination method, quantitatively explores the dechlorination mechanism of “active” CuCl by using a powder pressing method to prepare samples. The impacts of solution concentration and current density on the dechlorination are examined to reveal changes in the CuCl layer and assess chloride ion removal quantitatively.

## 2. Methods

### 2.1. Material and Experimental Procedure

The following experiments were designed based on the dechlorination conditions. Under both oxygen-rich and oxygen-poor conditions, NaOH solutions with pH levels of 8, 10, and 12 were used as electrolytes. Each test received 4 mL and 12 mL of a NaOH desalination solution, respectively. The experimental parameters are listed in Table 1. Each group used 1 g of CuCl powder, and the treatment time was 24 h. After soaking, the final products were analyzed using X-ray Diffraction (XRD) measurements, with CuKα radiation over a scan range of 10~90°. A phase identification and quantitative analysis were conducted using MDI Jade 6.5 software, with peaks calibrated for the CuCl (111), Cu_2_O (111), and Cu_2_(OH)_3_Cl (−101).

**Figure 1:**
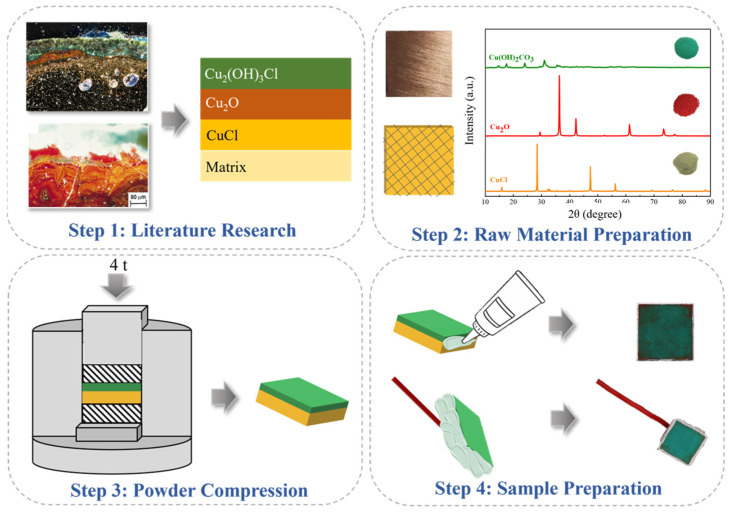
Procedures for preparing simulated samples of bronze with patina.

In a previous study, we investigated structural characteristics and the classification of patina on ancient bronze. A statistical analysis was carried out on the bronze corrosion product layer research in the past 20 years, and according to their composition and structure, the ancient bronze rust layer profiles were divided into four basic types: a chlorine-containing layer (Type I), oxide layer (Type II), hydrated layer (Type III), and tin-rich layer (Type IV) [27]. Ancient bronze with a chlorine-containing layer (Type I) is characterized by CuCl layers, Cu_2_O layers, and hydrated corrosion product layers, and faces severe secondary corrosion risk due to active chloride. Thus, considering the profile of the chlorides containing bronze corrosion product layers and focusing on the removal treatment of CuCl, a simulation sample was modeled as a structure containing CuCl, Cu_2_O, and Cu_2_(OH)_2_CO_3_. Here, a powder compressing method was used to prepare quantifiable samples referred to a typical archeological bronze sample, using 1 g of rust powder for each layer (for preparation details, see Figure 1 and [28]).

The samples were immersed in 400 mL of NaOH solutions for a desalination of 24 h. The NaOH concentrations were established at pH 8, pH 10, and pH 12 to explore the relationship between solution concentration and desalination effectiveness. Then, the experimental setup depicted in Figure S1 was established to examine the effect of current density on the dechlorination process. A DC constant-current supply (Corrtest CS1002, Wuhan, China) was used to supply a current for dechlorination. Using the sample as the cathode and a platinum sheet as the anode, a pH = 8 NaOH solution served as the electrolyte. Taking the pH = 8 NaOH immersion experiment result above as the blank group, the current densities of 2.5 μA/cm^2^, 12.5 μA/cm^2^, and 25 μA/cm^2^ were applied for 24 h, corresponding to potentials of approximately 1.27 V, 1.54 V, and 1.56 V vs. SCE.

### 2.2. Composition and Microstructure Characterization

The dechlorinated samples were embedded in epoxy resin and cut to expose their cross-sections. Then, the samples were gradually polished with SiC sandpaper up to 4000 grits, followed by cleaning with deionized water, drying, and gold sputtering. The composition was analyzed using a Tescan Vega III 3 XMU scanning electron microscope (SEM) (Xiamen, China) equipped with an energy-dispersive spectrometer (EDS) and a set of automatic measurement software (BPMA 1.0) for mineralogical parameters. Subsequently, the Tescan Vega III SEM (Brno, Czech Republic) and Bruker XFlash EDS (Billerica, MA, USA) were used to analyze the cross-sectional structure and elemental distribution, with an acceleration voltage of 15 kV. Rust layer thickness was measured using Image J 6.5 software, with three measurements taken at different positions of the CuCl layer on each sample to gain the average value.

### 2.3. NaOH Solution System Measurements

After desalination treatments, the 400 mL solution was diluted with deionized water to a final volume of 500 mL. The chloride ion content in the solution was analyzed using a Thermo Scientific ICS-5000 ion chromatograph (Waltham, MA, USA). Each NaOH solution pH level was measured three times to ensure result validity.

## 3. Results and Discussion

### 3.1. NaOH Promoting CuCl Powder Conversion

**Figure 2:**
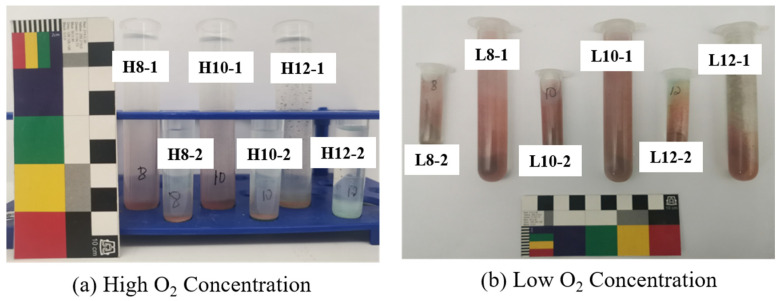
The final CuCl powder product after immersion in the NaOH solution for 24 h.

The NaOH treatment converts CuCl into more stable products while achieving desalination. To explore the transformation mechanism, CuCl powder was soaked in NaOH solutions to assess how product composition and quantity influenced by three factors, pH, O_2_ concentration, NaOH solution volume. Figure 2 shows the final products after soaking CuCl powder for 24 h in various NaOH conditions (detailed observations at different soaking times in Supplementary Figures S2 and S3). The products primarily consist of red and green powders, while no red products were observed in the H12-2 (pH = 12, 4 mL NaOH solution). CuCl is green, and the presence of the red product indicates that some of the CuCl was likely converted to Cu_2_O (red). Notably, small amounts of black products are observed at all pH = 12 groups besides the red and green powder, suggesting that high-pH conditions may alter the rust color during dechlorination.

**Figure 3:**
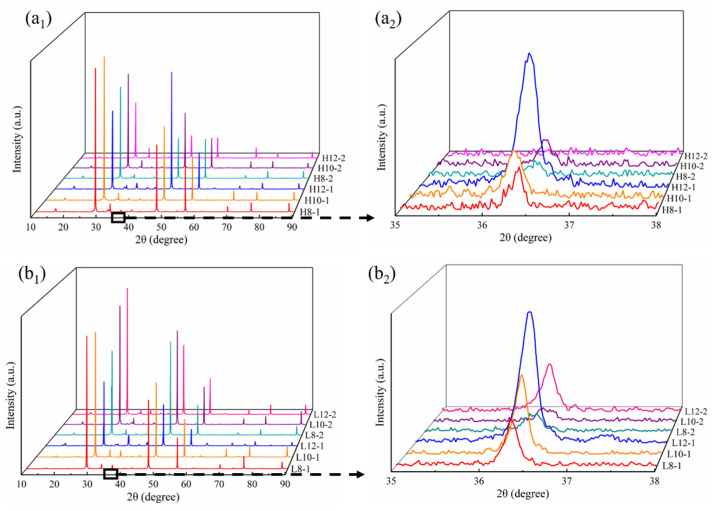
XRD patterns of the final CuCl powder product after immersion in the NaOH solution for 24 h; (**a_1_**) high-O_2_-concentration groups, (**a_2_**) characteristic peak of Cu_2_O in high-O_2_-concentration groups, (**b_1_**) low-O_2_-concentration groups, (**b_2_**) characteristic peak of Cu_2_O in low-O_2_-concentration groups.

The final powder was analyzed by XRD to identify the composition of the CuCl transformation products. As shown in Figure 3(a_1_,b_1_), the components of the CuCl transformation products are CuCl, Cu_2_O, and Cu_2_(OH)_3_Cl, with characteristic peaks at 28.5°, 36.4°, and 16.2°, respectively [3,29,30,31,32]. According to XRD patterns, CuCl may undergo the reactions as described in Equations (1) and (2) [3,6].

2CuCl + 2OH^−^ → Cu_2_O + H_2_O + 2Cl^−^ (1)

CuCl + O_2_ + 4H_2_O → 2Cu_2_(OH)_3_Cl + 2H^+^ + 2Cl^−^ (2)

Previous studies showed that various intermediate reactions may occur during the CuCl transformation process [33,34,35,36,37], such as Equation (3) [38,39], Equation (4) [30], and Equation (5) [38,39].

CuCl + Cl^−^ → CuCl_2_^−^ (3)

CuCl_2_^−^+ 2OH^−^ → Cu_2_O + H_2_O + 2Cl^−^ (4)

4CuCl_2_^−^ + O_2_ + 4H_2_O → 2Cu_2_(OH)_3_Cl + 2H^+^ + 6Cl^−^ (5)

Despite having the same reactants, different product combinations may arise due to differences in reaction conditions. For instance, in Figure 2, H12-1, the absence of red-colored products in the final transformation products is observed. This phenomenon is further confirmed by the XRD spectrum shown in Figure 3(a_2_), with no characteristic peak of Cu_2_O at 36.4°. Apart from the composition characteristics in H12-2, we previously mentioned the presence of black substances at pH = 12 groups in Figure 2. However, no corresponding characteristic peaks are identified as black particles in Figure 3, possibly because their content is below the detection limit of XRD (typically 5 wt.%). And the qualitative result of them is presented later.

**Figure 4:**
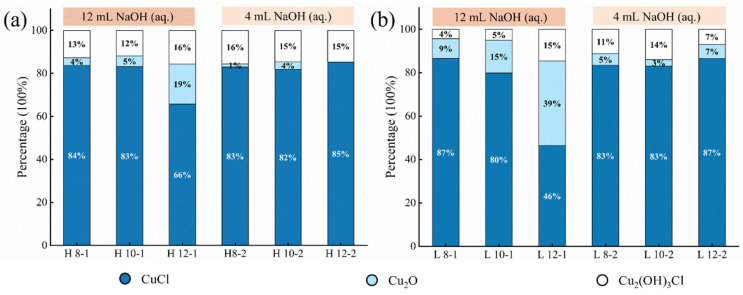
Quantitative results of the final product after immersing CuCl powder in a NaOH solution for 24 h according to XRD spectra; (**a**,**b**): high-O_2_-concentration groups, low-O_2_-concentration groups.

Each group contained 1 g of CuCl, allowing for a quantitative analysis of XRD data to elucidate the relationships between parameters and product transformation. Figure 4a,b depict the influence of oxygen content differences on the composition ratio of CuCl transformation products. Results show that the conversion rate of CuCl in the low-O_2_-concentration groups is generally higher than that in high ones, with a higher proportion of Cu_2_O generated. No oxygen is required to generate Cu_2_O, according to Equations (1) and (2). Thus, Cu_2_O formation is more favorable under low-oxygen dechlorination conditions. The difference in solution volume between 12 mL and 4 mL simulates the effect of varying desalination solution contents in rust layer environments on CuCl removal. Results in Figure 4 show that the conversion rate of CuCl is higher with 12 mL solution volumes compared to lower ones under the same pH conditions, indicating that an insufficient solution in CuCl rust layers may inhibit the removal of CuCl. As the volume is 12 mL, the conversion rate of CuCl increases with an increasing pH value. However, the pattern of the CuCl conversion rate is not significant when the volume is 4 mL, indicating insufficient ability to resist changes.

**Figure 5:**
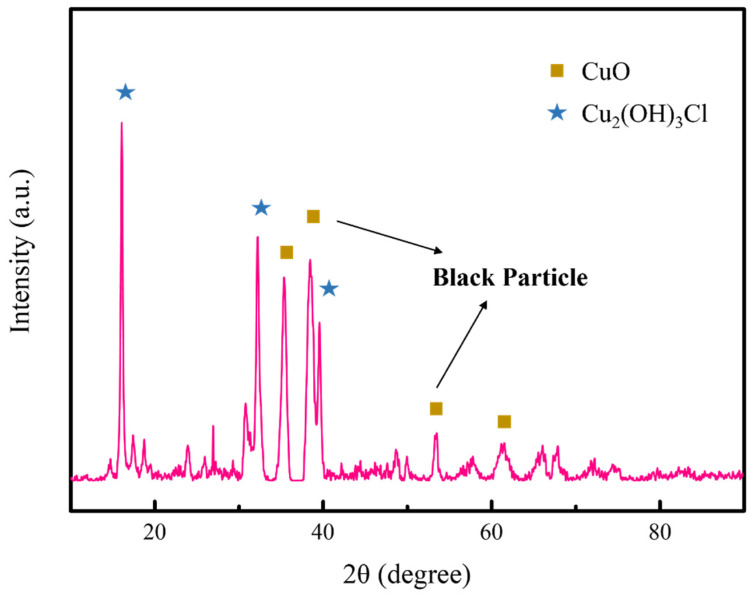
XRD patterns of black particles in pH = 12 groups.

According to the XRD patterns in Figure 5, the black substance mainly consists of characteristic peaks at 35.4° corresponding to CuO and at 16.2° to Cu_2_(OH)_3_Cl. Since Cu_2_(OH)_3_Cl is mostly a green color while CuO appears as black, the products formed from CuCl conversion under pH = 12 conditions should be CuO. Its formation is favored under higher-pH conditions confirmed by the Cu-Cl-H_2_O Pourbaix diagram (E–pH) [40]. It can form through several different ways, Equation (6) [41] and Equation (7) [40].

2Cu_2_O + O_2_ → 4CuO (6)

Cu_2_O + H_2_O → 2CuO + 2H^+^ + 2e^−^ (7)

A common consensus reported by the literature was Cu_2_O as a mediator to transform into Cu(II) (primarily CuO or Cu(OH)_2_) [39]. Some studies also indicate that NaOH solution dechlorination produced CuO in the rust layer [16], but its formation would bring the discoloration of the rust layer and further change the original appearance of bronze.

### 3.2. Composition and Microstructure of Rust Layer

**Figure 6:**
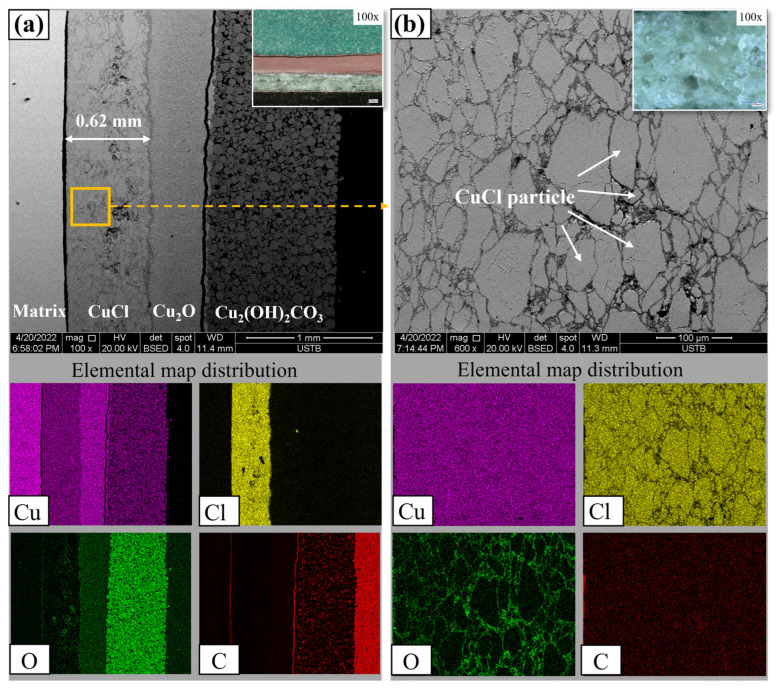
Structural and elemental map distribution of simulated sample; (**a**) cross-section of layer, (**b**) CuCl layer.

Previous studies used archeological or corrosion simulated samples; it was challenging to compare changes in rust layer composition and structure due to the complex layering of corrosion products. To address this issue, we employed a powder compressing method to ensure that both the weight of powder and the layer thickness of samples were consistent for studying dechlorination mechanisms. The composition and structure of the sample are shown in Figure 6. It consists of three layers from an outer to inner order, Cu_2_(OH)_2_CO_3_, Cu_2_O, and CuCl, with the CuCl layer thickness of approximately 0.62 mm (Figure 6a). Figure 6b shows a magnified micrograph of the CuCl layer, with white arrows indicating CuCl grains (pale gray partly with green).

#### 3.2.1. Effect of Solution Concentration

**Figure 7:**
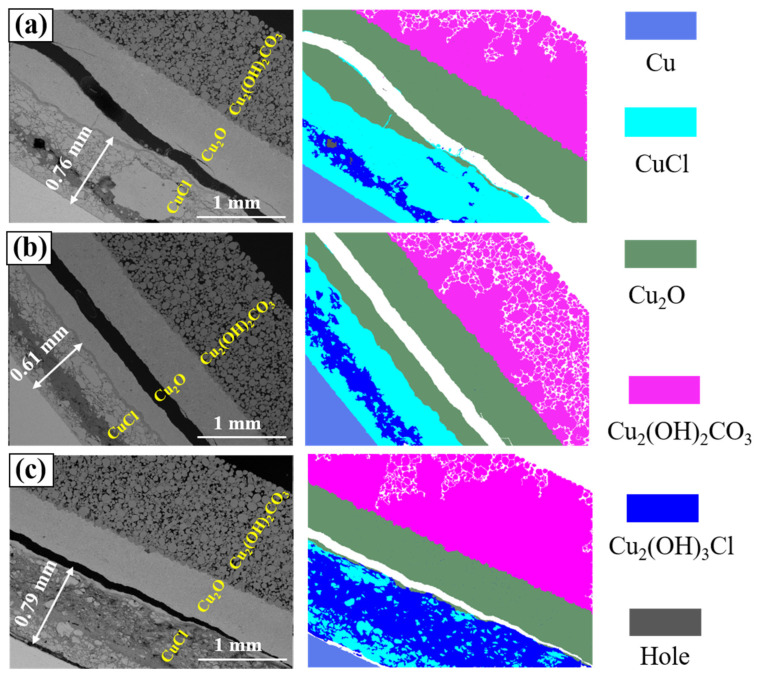
The cross-sectional composition and structure of rust layers treated with a NaOH solution at different concentrations; (**a**–**c**): pH = 8, pH = 10, pH = 12.

Figure 7 illustrates cross-sectional characteristics of rust layers after dechlorination with different solution concentrations. It is observed that Cu_2_O and Cu_2_(OH)_2_CO_3_ layers show no significant changes, whereas the product of CuCl layer transformation is identified as Cu_2_(OH)_3_Cl. This indicates that the dechlorination solution penetrates the CuCl layer interior, effectively reacting with it to form Cu_2_(OH)_3_Cl. The CuCl layers at pH = 8 and pH = 12 (Figure 7a,c) both exceeded 0.7 mm in thickness, representing an approximately 0.1 mm increase compared to untreated samples (Figure 6a). This expansion of the rust layers correlates with density: CuCl has a density of 4.1 g/cm^3^, whereas formed Cu_2_(OH)_3_Cl (density: 3.5 g/cm^3^) is less dense than CuCl, contributing to expansion. However, the sample at pH = 10 does not exhibit noticeable expansion, possibly due to insufficient CuCl conversion to alter thickness (Figure 7b). Correspondingly, further solution measurements confirm the lower CuCl conversion rate in the pH = 10 group, showing that the chloride ion removal at pH = 10 is lower compared to samples at pH = 8 and pH = 12. This phenomenon might be the result of both pH = 8 and pH = 10 having a lower solution concentration and an experimental error.

#### 3.2.2. Effect of Current Density

**Figure 8:**
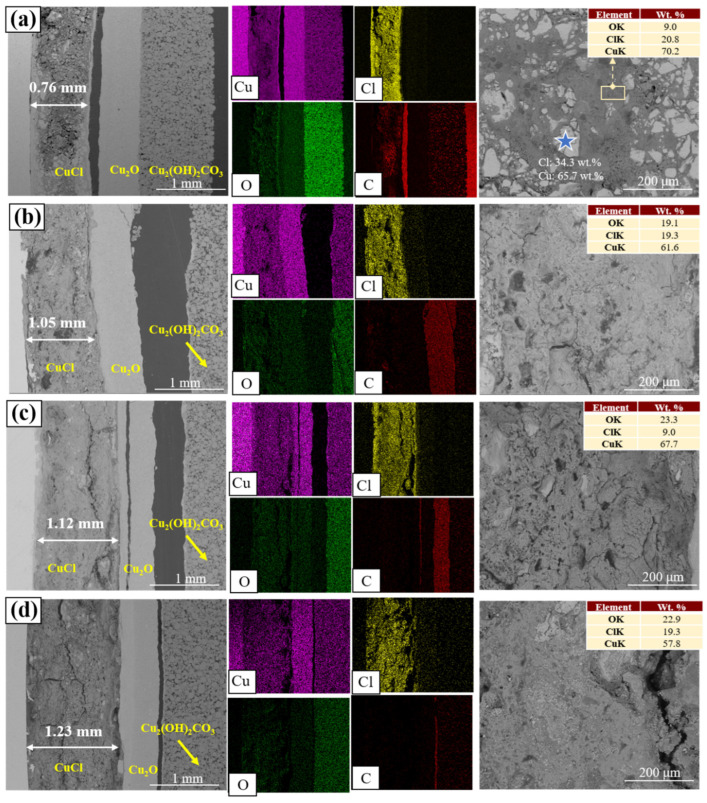
SEM-EDS analysis of cross-section of sample after constant-current NaOH dechlorination treatment; (**a**–**d**): 0 μA/cm^2^, 2.5 μA/cm^2^, 12.5 μA/cm^2^, 25 μA/cm^2^.

Electrochemical treatments were conducted to compare the effects of different current densities on the change in rust layers. CuCl and Cu_2_(OH)_3_Cl are supposed to be the main composition of the CuCl layer based on the previous analysis of the composition and color of the conversion product (Figure S4). Confirmed by EDS results, it can be determined that the oxygenated zone should be Cu_2_(OH)_3_Cl (the O wt.% is approximately 22.4%), while the remaining part is CuCl (marked as blue star in Figure 8a, Cl: 34.3 wt.% and Cu: 65.7 wt.%). This indicates that the electrochemical treatment did not alter the mechanism of CuCl removal, converting into Cu_2_(OH)_3_Cl. Figure 8a shows the sample treated without an electrical current, and blue markings point out unconverted CuCl particles. However, after electrochemical treatments, CuCl grains are entirely replaced by Cu_2_(OH)_3_Cl (Figure 8b–d), demonstrating that these treatments facilitate a more thorough conversion of CuCl. The thickness of CuCl rust layers was measured by Image J software and it was found that the thickness increased to 1.05 mm, 1.12 mm, and 1.23 mm with increasing current density, respectively. These results indicate that applying an electrical current promotes the conversion of CuCl, leading to thicker rust layers. This phenomenon further supports that an increased current density enhances the dechlorination reaction. A previous study already reported objects’ spalling phenomenon and took bubble formation as a possible contributor [20]. Therefore, attention should be paid to whether fragility or cracking may be related to less dense corrosion products, rather than simply attributing it to gas evolution caused by an electrical process.

### 3.3. Change in NaOH Dechlorination Solution System

CuCl removal is crucial to suppress ‘secondary’ corrosion, yet quantifying the removal of chlorine from CuCl remains challenging. A conventional study periodically monitored chloride ion levels in dechlorination solutions and determined the endpoint of dechlorination using AgNO_3_ titration or by difficult-to-detect chloride ions in a solution [23,26]. However, conventional samples often exhibited variations in chlorine content and complexity in chlorine sources, making it difficult to figure out the source and removal rate. Therefore, during the preparation of powder compressed samples, CuCl was fixed at 1 g (Cl: 0.3581 g. Mr_(CuCl)_ = 99 g/mol, Mr_(Cl)_ = 35.45 g/mol) to quantitatively study dechlorination. Meanwhile, to ensure that all chlorine remained in the sample–solution system, the solutions’ dechlorination were not changed during the process.

#### 3.3.1. Effect of Solution Concentration

**Figure 9:**
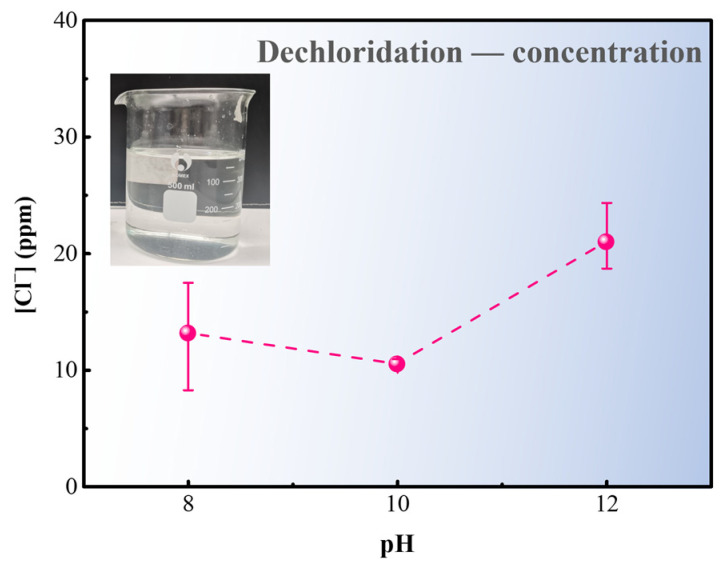
Chloride ion concentration in dechlorination solution after immersion in NaOH solution with different concentration.

The inset photograph in Figure 9 shows no precipitation or turbidity in the solution after dechlorination, indicating that chloride is in a dissolving state. Hence, the total amount of chlorine removed can be determined by analyzing using ion chromatography. IC results show that measurements of the chloride ion concentrations are 13.14 ppm, 10.49 ppm, and 20.97 ppm. Assuming that all amount of CuCl in the rust layer is converted to Cu_2_(OH)_3_Cl completely, and released Cl is transferred into the desalination solution. The total chloride ion content in the NaOH solution should be 0.1791 g (500 mL) based on the correspondence between Cu and Cl. Thus, as the measured chloride ion concentration reaches 358.2 ppm, it represents the endpoint (c_0_) of dechlorination in this system. If c_t_ represents the measured chloride ion concentration at a specific time, the progress of desalination E_t_ at that time can be expressed as

E_t_ = c_t_/c_0_ × 100% (8)

According to Equation (8), the calculation results of E_t=24h_ are approximately 3.7%, 3.0%, and 5.9%, respectively, indicating that all of them are still in initial stages. In Figure 9, the dechlorination progress is approximately twice as fast with a NaOH solution concentration of 10^−2^ M (pH = 12) compared to the 10^−4^ M (pH = 10). This suggests that increasing the concentration of solutions accelerates the conversion and removal of CuCl. However, the curve in Figure 9 shows that the increase in concentration does not correlate positively with the rate of chloride ion removal.

#### 3.3.2. Effect of Current Density

**Figure 10:**
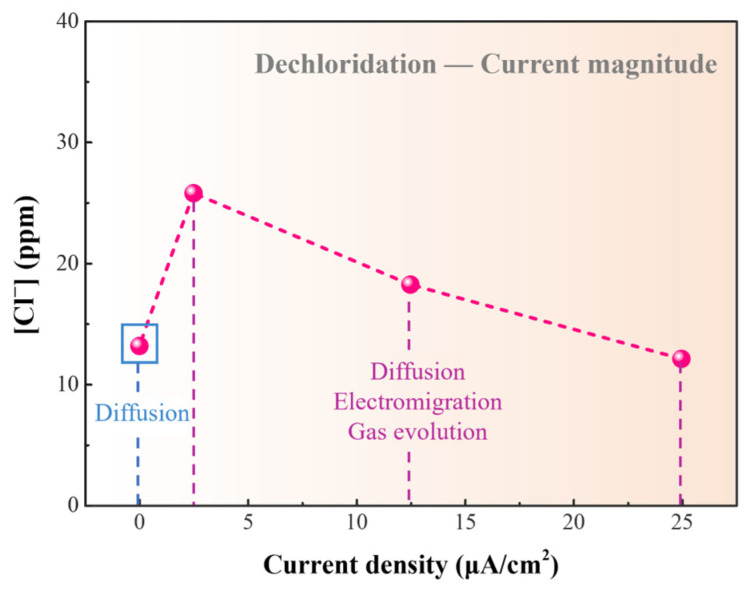
Chloride ion concentration in dechlorination solution after immersion in NaOH solution with different current densities.

Figure 10 shows the results of chloride concentration in the dechlorination solution after applying electrical currents ranging from 0 μA/cm^2^ to 25 μA/cm^2^ for 24 h. With increasing current density, there is an initial increase followed by a decrease in chloride ion concentration. According to Figure 9, the chloride ion concentration without an electrical current is 13.14 ppm. When the current density is increased to 2.5 μA/cm^2^, the removal of chloride ions doubles compared to 0 μA/cm^2^. This indicates that the electric field promotes the migration of chloride ions, shifting the mass transfer mechanism from diffusion to diffusion and electromigration. Electromigration is a more efficient method for ion mass transfer compared to diffusion. It is noteworthy that after reaching a current density of 12.5 μA/cm^2^, the chloride ion concentration in the solution starts to decrease. Even at a current density of 25 μA/cm^2^, the chloride ion content drops to 12.07 ppm. This phenomenon may be attributed to some chloride ions being oxidized to chlorine gas at the anode and removed. Given that the standard electrode potential for Cl_2_/Cl^−^ is 1.36 V, and for O_2_/H_2_O, it is 1.229 V, chloride ions are more prone to lose electrons and be oxidized into chlorine gas than water. Therefore, some chloride ions may be released in the form of gas, resulting in a decrease in chloride ion concentration in the solution. Due to the possibility of chloride ions being released as gas besides existing as chloride ions after CuCl releases chlorine, the measured value of c_t_ in Equation (8) cannot be replaced by the ion measurement value. Therefore, Equation (8) is not suitable for evaluating the dechlorination progress in an electrified system.

## 4. Conclusions

1. A new sample simulation method was developed to address challenges of quantitative studies from traditional experimental samples, providing an effective method to evaluate dechlorination methods.
2. The NaOH desalinization method achieves dechlorination by converting CuCl to Cu_2_(OH)_3_Cl, but this transformation leads to an expansion of the rust layer. The thickness of the CuCl layer at a current density of 25 μA/cm^2^ is nearly doubled.
3. The analysis of the solutions proves that adjusting the concentration of reactants and current density can significantly improve the effectiveness of dechlorination. Both can potentially double the chloride removal rates.
4. Based on the CuCl input data and transformation product analysis, the theoretical endpoint (c_0_) and CuCl dechlorination progress (E) can be calculated accurately. However, further calculation and experimental methods could be designed to quantify dichlorination progress under electrolysis conditions.

## Figures and Tables

**Table 1:** Experimental parameters for NaOH promoting CuCl powder conversion.

pH		8	10	12
High oxygen	12 mL	H8-1	H10-1	H12-1
	4 mL	H8-2	H10-2	H12-2
Low oxygen	12 mL	L8-1	L10-1	L12-1
	4 mL	L8-2	L10-2	L12-2

## Data Availability

The original contributions presented in this study are included in the article/Supplementary Material. Further inquiries can be directed to the corresponding author.

## Supplementary Materials

The following supporting information can be downloaded at: https://www.mdpi.com/article/10.3390/ma17246126/s1, Figure S1. Schematic diagram of electrified dechlorination device. Figure S2. CuCl powder conversion immersed in high-oxygen group in NaOH solution during 24 hours. Figure S3. CuCl powder conversion immersed in low-oxygen group in NaOH solution during 24 hours. Figure S4. Structure and color of samples after electrochemical dechlorination treatments.
