# Supplementary material for: Revisiting Mechanism of NaOH Dechlorination Treatments for Bronze Conservation in Quantitative Study
Source: Materials (Basel). 2024 Dec 14;17(24):6126. doi: 10.3390/ma17246126 (PMC11678902; doi:10.3390/ma17246126)
Supplement: Supplementary file 1 [file materials-17-06126-s001.zip › materials-3348991-supplementary.pdf]

**Supplementary Information for**

# Revisiting mechanism of NaOH dechlorination treatments for bronze conservation in quantitative study

**X. Yang et al.**

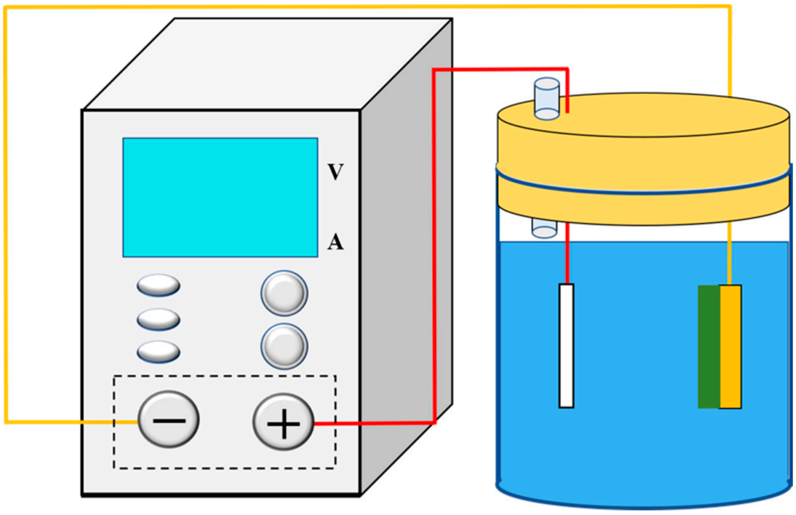


**Figure S1. Schematic diagram of electrified dechlorination device.**

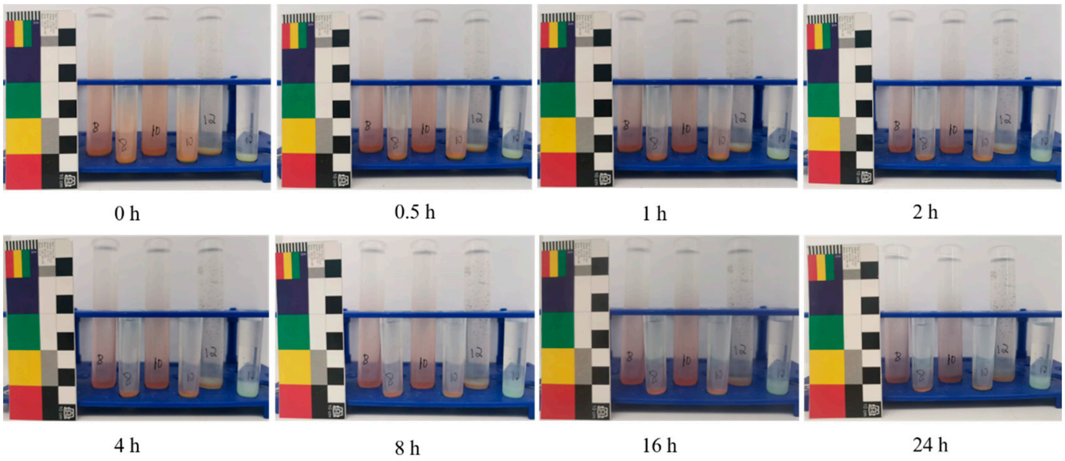


**Figure S2. CuCl powder conversion immersed in high-oxygen group in NaOH solution during 24 hours.**

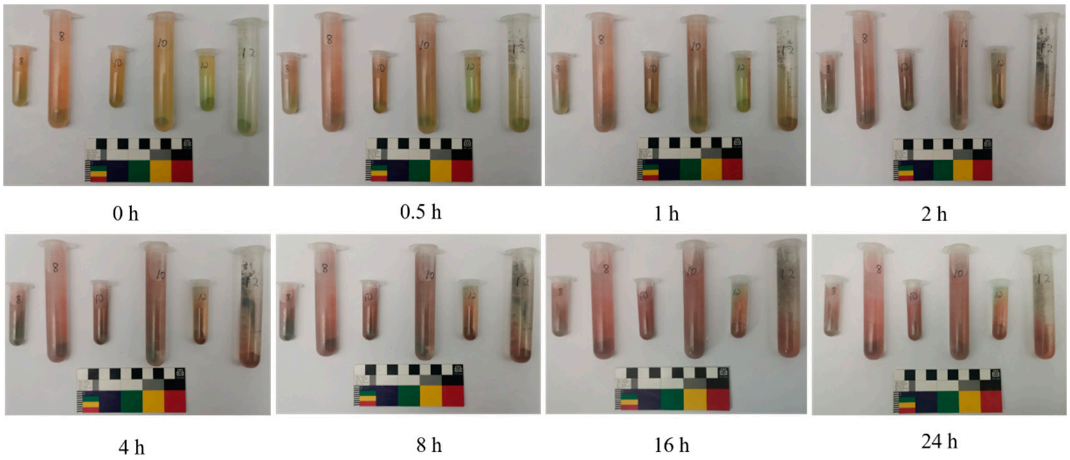


**Figure S3. CuCl powder conversion immersed in low-oxygen group in NaOH solution during**

**24 hours.**

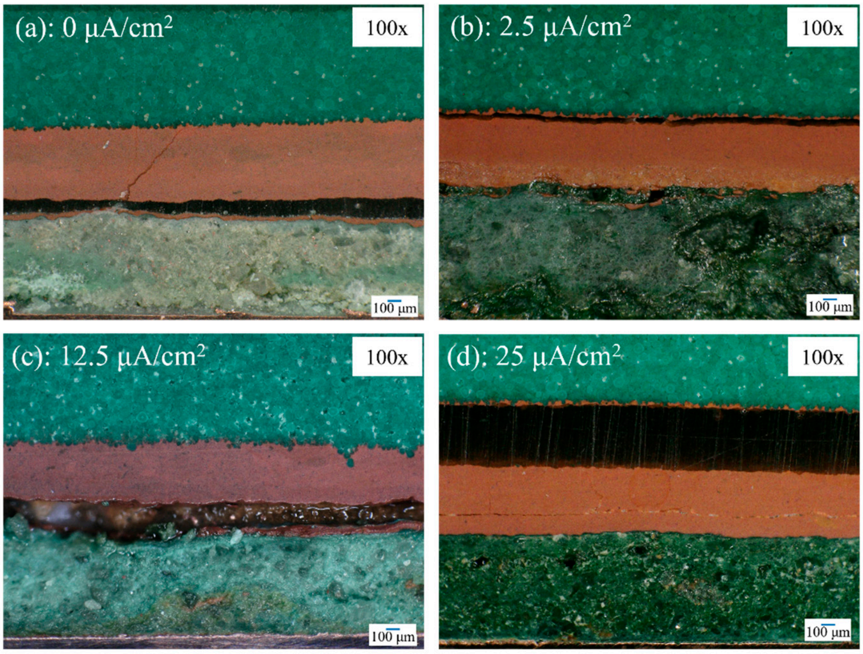


**Figure S4. Structure and color of samples after electrochemical dechlorination treatments.**
